# Epidemiological Trends in Mortality From Acute Myocardial Infarction With Essential and Secondary Hypertension: CDC WONDER Database Analysis, 2000–2024

**DOI:** 10.1002/clc.70420

**Published:** 2026-07-18

**Authors:** Javeria Akhter, Waleed Ahmad, Aasiya Shahbaz, Aniqa Dejwani, Nafila Zeeshan, Jamil Nasrallah

**Affiliations:** ^1^ Indus Hospital and Health Network Karachi Pakistan; ^2^ Lahore Medical and Dental College Lahore Pakistan; ^3^ Dow University of Health Sciences Karachi Pakistan; ^4^ Department of Medicine, Faculty of Medical Sciences Lebanese University Beirut Lebanon

**Keywords:** acute myocardial infarction, CDC wonder, hypertension, mortality, United States

## Abstract

**Background:**

Acute myocardial infarction (AMI) with essential hypertension (EH) and secondary hypertension (SH) remains a leading cause of mortality. Despite the overall decline, disparities in mortality trends across age group, gender, urbanization, racial, and regional subgroups remain underexplored.

**Methods:**

Mortality data from the CDC WONDER (2000–2024) were analyzed for AMI (ICD‐10 I21) as the primary cause of death and EH (ICD‐10 I10) and SH (ICD‐10 I15.0, I15.9) as a contributing factor in individuals aged ≥ 25 years. Age‐adjusted mortality rates (AAMRs) were standardized to the US 2000 population. Joinpoint regression estimated annual percentage changes (APCs) with 95% confidence intervals (CIs).

**Results:**

AMI with hypertension caused 842,646 deaths, with AAMR declining from 17.63 in 2000 to 12.26 in 2024. Overall, the AAMR showed a significant decline across the study period (AAPC: −1.71%). Males consistently had a higher mean AAMR (18.50) than females (11.82). Upward inflections were noted in the South region during 2018–2021 (APC: 11.17%; *p* = 0.004), Non‐Hispanic Black individuals during 2018–2021 (APC: 9.45%; *p* = 0.053), metropolitan areas during 2018–2020 (APC: 7.71%; *p* = 0.038), and among adults aged 65–74 years during 2018–2021 (APC: 7.15%; *p* = 0.039). State‐level variations were observed.

**Conclusion:**

Despite the overall decline, rising mortality trends were observed in the Southern region (2018–2021), metropolitan areas, and among individuals aged 65–74 years, highlighting emerging patterns and emphasizing the need for targeted interventions.

## Graphical abstract

Age‐adjusted mortality rate from acute myocardial infarction with hypertension declined in the United States from 2000 to 2024. However, increasing mortality in the Southern region, metropolitan areas, and adults aged 65–74 years highlights persistent disparities and the need for targeted prevention and management strategies.

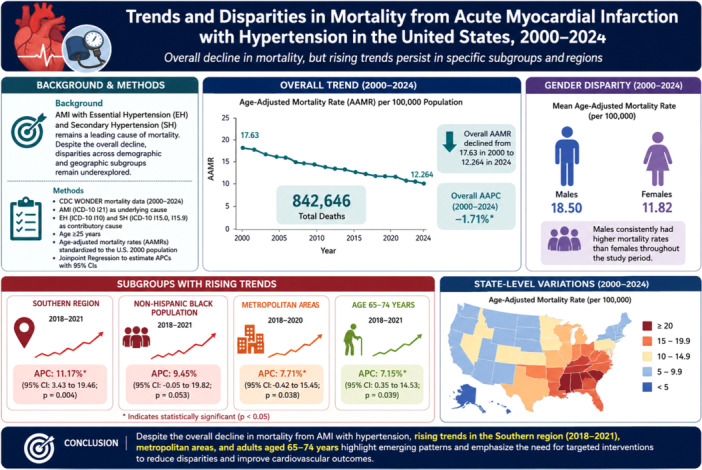

## 1 Introduction

Cardiovascular disease (CVD) is one of the leading causes of mortality, with acute myocardial infarction (AMI) being a major contributor, reporting over 1 million deaths each year in the United States (US) [1]. Hypertension—categorized as essential (primary) or secondary—is one of the most important modifiable risk factors for AMI, contributing to myocardial strain, vascular damage, and an increased risk of ischemic events [2].

In the US, approximately 116 million (1 in 2) adults are affected by hypertension, contributing to substantial health and economic burden, yet control rates remain at only about one in four [3]. Essential hypertension (EH) accounts for the majority of cases, while secondary hypertension, although less prevalent, is often linked with more severe outcomes due to underlying endocrine disorders [4]. Both forms of hypertension significantly increase the risk of adverse cardiac events, including AMI.

The association between hypertension and AMI presents a serious health concern. Multiple studies have constantly shown a significant increase in mortality associated with hypertension, with AMI being a primary driver of that excess risk [5, 6]. Studies have reported that approximately 50%–60% of patients hospitalized with Acute coronary syndromes (ACS) have a history of hypertension [7]. Hypertensive patients who suffer an AMI also face a higher risk of death in the years following the event compared to normotensive patients. Notably, patients with a history of hypertension who experience ST‐elevation myocardial infarction (STEMI) have significantly higher in‐hospital and 30‐day mortality rates, even after adjusting for other risk factors [8].

Despite medical advancements and public health efforts, disparities in hypertension management and AMI outcomes persist across population subgroups and regions, including by sex, race and ethnicity, and geography. This study aims to assess trends in AMI‐related deaths among adult patients with essential and secondary hypertension in the United States from 2000 to 2024, with a focus on identifying sex‐based, racial and ethnic, and regional disparities.

## 2 Methods

### 2.1 Study Design and Database

A cross‐sectional study was performed to assess the AMI‐associated mortality rate in patients with essential and secondary hypertension as a comorbidity in the US from 2000 to 2024. Mortality data was sourced from Centers for Disease Control and Prevention WONDER (Wide‐Ranging Online Data for Epidemiologic Research) database, focusing on adult hypertensive patients (age > 25) who died of AMI. Our analysis specifically used the Multiple Cause‐of‐Death Public Use Record database to identify cases where AMI was listed as the underlying cause and hypertension as the contributing cause of mortality on death certificates across the US from 2000 to 2024 [9]. Institutional review board approval was not required, as CDC WONDER contains anonymized, publicly available data. This study followed the Strengthening the Reporting of Observational Studies in Epidemiology (STROBE) guidelines for cross‐sectional studies [10]. Deaths were identified using ICD‐10 codes I10 for EH, I15 for SH, and I21 for AMI [11, 12]. For this study, essential and secondary hypertension were analyzed collectively as a single hypertension category, representing hypertension as a contributing cause of death. Subgroup analysis by hypertension type was performed; however SH‐related AMI mortality could not be analyzed independently due to suppressed or zero death counts in the CDC WONDER database.

### 2.2 Data Abstraction

Data extracted for analysis included gender, race, age group, year, urbanization, census region, and place of death from 2000 to 2024. Sex was categorized as male and female. Race and ethnicity groups were classified as non‐Hispanic (NH) American Indian or Alaska Native, NH Asian or Pacific Islander, NH Black or African American, NH White, and Hispanic or Latino. Age was categorized into seven 10‐year groups: 25–34, 35–44, 45–54, 55–64, 65–74, 75–84, and 85+ years. The population was divided into metropolitan (large central, large fringe, medium, and small) and non‐metropolitan (micropolitan and noncore) categories according to the 2013 US Census urbanization classification [13]. Urbanization data are presented through 2020 only, as the NCHS revised its urban–rural classification scheme in 2021, introducing a structural discontinuity that precludes direct comparability with prior years. Census regions were classified as Northeast, Midwest, South, and West based on US Census Bureau definitions. For subgroup analyses, data were additionally extracted for deaths where EH (I10) was specifically listed as a contributing cause.

### 2.3 Statistical Analysis

Crude mortality rates (CMR) and age‐adjusted mortality rates (AAMR) for hypertension and AMI‐associated deaths were calculated. The CMR was determined by dividing the number of deaths by the corresponding US population, while the AAMR accounted for variations in age distribution, facilitating standardized comparisons across groups over time. The US population as of 2000 was used as the standard population for AAMR calculations [14]. We used the Joinpoint Regression Program (version 5.2.0, National Cancer Institute), which applies a segmented log‐linear modeling approach to identify significant changes in mortality trends over time [15]. For overall trends, a maximum of four Joinpoints was permitted; for subgroup analyses, a maximum of three Joinpoints was used given smaller cell counts. A minimum of three observations was required between Joinpoints. Model selection was based on a permutation test with 4499 permutations at a significance level of α = 0.05. For each trend, we report the annual percent change (APC) and the average annual percent change (AAPC), along with their 95% confidence intervals (CI). An APC was considered increasing or decreasing if the slope describing the change in mortality was significantly different from zero, based on two‐tailed *t*‐testing. Statistical significance was set at *p* ≤ 0.05.

## 3 Results

A total of 842,646 AMI deaths related to hypertension occurred in people aged ≥25 years between 2000 and 2024. Among these cases, 35.3% occurred in the descendant's home, 25.6% in inpatients, and 20.09% in outpatients (Supporting Information S1: Table 1).

### 3.1 Annual Trends in AAMR for Essential and Secondary Hypertension‐Related Acute Myocardial Infarction

**Figure 1:**
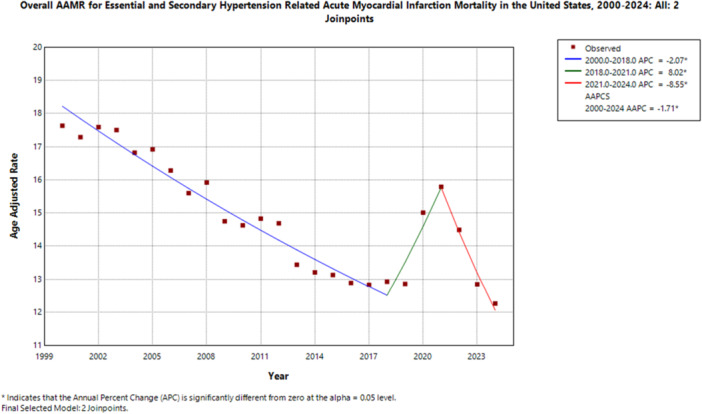
Overall age‐adjusted mortality rate for essential and secondary hypertension‐related acute myocardial infarction in the United States, 2000–2024.

For individuals aged 25 years and older, the AAMR for AMI related to essential and secondary hypertension was 17.63 in 2000 and decreased to 12.26 in 2024. From 2000 to 2018, the AAMR showed a significant downward trend, with an APC of −2.07% (*p* < 0.05). This was followed by a sharp reversal from 2018 to 2021, during which the AAMR increased significantly, with an APC of 8.02% (*p* < 0.05), peaking around 2021. From 2021 to 2024, the AAMR declined again significantly, with an APC of −8.55% (*p* < 0.05). Over the full study period from 2000 to 2024, the AAMR demonstrated an overall significant downward trend, with an AAPC of −1.71% (*p* < 0.05) (Supporting Information S1: Table 2; Figure 1).

### 3.2 Sex‐Stratified AAMR for Essential and Secondary Hypertension‐Related Myocardial Infarction

**Figure 2:**
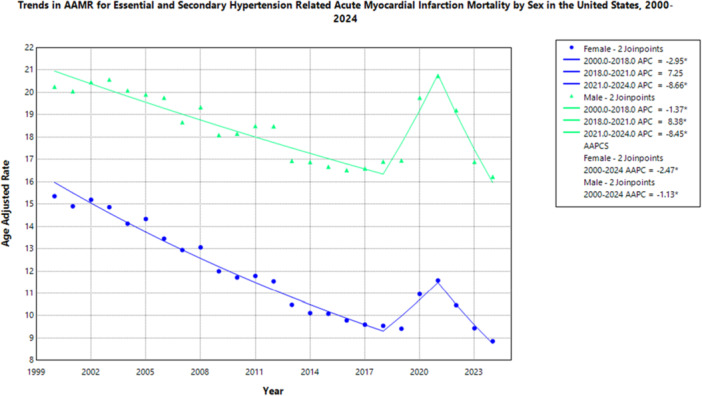
Trends in age‐adjusted mortality rates for essential and secondary hypertension‐related acute myocardial infarction by sex in the United States, 2000–2024.

Throughout the study, males had consistently higher AAMRs than females (male: 18.5; female: 11.8). Male AAMR was 20.25 (95% CI: 19.91–20.58) in 2000 and decreased significantly to 16.90 in 2018 (APC: −1.37; 95% CI: −1.62 to −1.12). This was followed by a significant increase, with AAMR rising to a peak of 20.73 in 2021 (APC: 8.38; 95% CI: 1.26 to 15.99), before decreasing again to 16.22 in 2024 (APC: −8.45; 95% CI: −11.48 to −5.32). Comparably, female AAMR was 15.35 in 2000 and it then showed a significant decline to 9.55 in 2018 (APC: −2.95; 95% CI: −3.20 to −2.70). A modest rebound occurred between 2018 and 2021, with AAMR peaking at 11.58, though this increase was not statistically significant (APC: 7.25; 95% CI: −1.18 to 16.39), followed by a significant decline to 8.86 in 2024 (APC: −8.66; 95% CI: −12.31 to −4.86) (Supporting Information S1: Table 3; Figure 2).

### 3.3 Geographic Trends in AAMR for Essential and Secondary Hypertension‐Associated MI

The AAMR varied significantly between the states, with Alaska having the lowest AAMR of 6.82 (95% CI: 6.03–7.65) and Mississippi having the highest AAMR of 37.58 (95% CI: 36.81–38.37). Mississippi, Arkansas, South Dakota, Kentucky, Rhode Island, and Tennessee were among the states in the top 90th percentile; their AAMR was substantially higher than that of the states in the bottom 10th percentile, which included Alaska, Nevada, Connecticut, Utah, Montana, and Minnesota (Supporting Information S1: Table 4).

**Figure 3:**
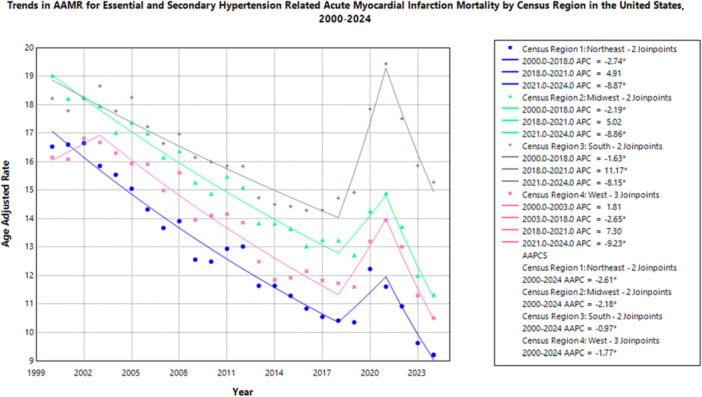
Trends in age‐adjusted mortality rates for essential and secondary hypertension‐related acute myocardial infarction by census region in the United States, 2000–2024.

Regionally, the Northeast showed a significant decline from 2000 to 2018 (APC: −2.74), followed by a non‐significant increase from 2018 to 2021 (APC: 4.91), and then a significant decline from 2021 to 2024 (APC: −8.87). Overall, the Northeast declined significantly from 2000 to 2024 (AAPC: −2.61). In the Midwest, there was a significant decline between 2000 and 2018 (APC: −2.19), followed by a non‐significant increase from 2018 to 2021 (APC: 5.02). Subsequently, there was a significant decrease between 2021 and 2024 (APC: −8.86). Similarly, the Southern region showed a significant decrease from 2000 to 2018 (APC: −1.63), followed by a significant increase between 2018 and 2021 (APC: 11.17), then a significant decline from 2021 to 2024 (APC: −8.15). In the Western region, the AAMR showed a non‐significant increase from 2000 to 2003 (APC: 1.81), followed by a significant decline from 2003 to 2018 (APC: −2.65); the subsequent period saw a non‐significant increase from 2018 to 2021 (APC: 7.30), followed by a significant decline from 2021 to 2024 (APC: −9.23) (Supporting Information S1: Table 5; Figure 3).

### 3.4 Urbanization‐Stratified AAMR for Essential and Secondary Hypertension‐Related Myocardial Infarction

**Figure 4:**
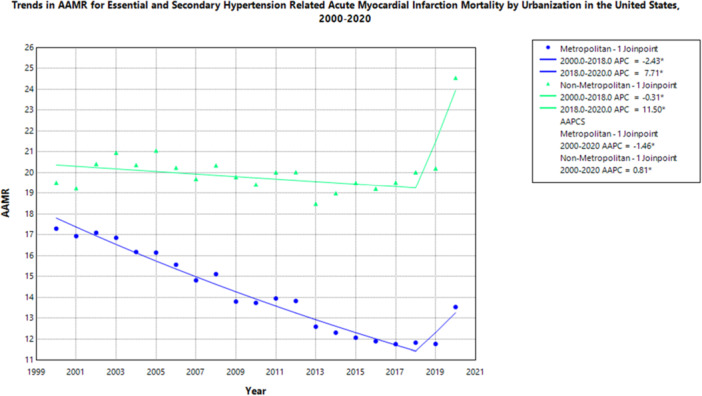
Trends in age‐adjusted mortality rates for essential and secondary hypertension‐related acute myocardial infarction by urbanization in the United States, 2000–2020.

Starting with AAMRs of 17.30 for metropolitan areas and 19.52 for non‐metropolitan areas in 2000, the AAMR for non‐metropolitan areas declined slightly but significantly to 20.02 in 2018 (APC: −0.31; 95% CI: −0.60 to −0.02), before rising sharply and significantly to 24.55 in 2020 (APC: 11.50; 95% CI: 2.93 to 20.79). Similarly, the AAMR of metropolitan areas declined significantly from 17.30 in 2000 to 11.83 in 2018 (APC: −2.43; 95% CI: −2.66 to −2.20), before increasing significantly between 2018 and 2020 to 13.54 (APC: 7.71; 95% CI: 0.46 to 15.49). Despite these fluctuations, metropolitan areas consistently maintained lower AAMRs than non‐metropolitan areas throughout the study period. Over the full study period, metropolitan areas showed a significant overall decline (AAPC: −1.46; 95% CI: −2.12 to −0.79), whereas non‐metropolitan areas showed a significant overall increase (AAPC: 0.81; 95% CI: 0.03 to 1.60) (Supporting Information S1: Table 6; Figure 4).

### 3.5 Age‐Adjusted Mortality Rates for Hypertension‐Related Myocardial Infarction Stratified by Race/Ethnicity

When race and ethnicity were taken into account, NH Black or African American individuals had the highest overall AAMR, followed by NH White, Hispanic or Latino, NH American Indian or Alaska Native, and NH Asian or Pacific Islander populations. The pooled AAMRs were as follows: NH Black or African American: 23.43, NH White: 14.22, NH American Indian or Alaska Native: 11.46, Hispanic or Latino: 12.64, and NH Asian or Pacific Islander (including the Asian‐only subgroup from 2021 onward): 10.14.

Among NH Black or African American individuals, the AAMR decreased significantly between 2000 and 2018 (APC: −3.61%; 95% CI: −3.91 to −3.30), followed by a borderline non‐significant increase through 2021 (APC: 9.45%; *p* = 0.053), and then a significant decline through 2024 (APC: −10.68%; 95% CI: −14.74 to −6.41). The overall AAPC from 2000 to 2024 was −2.99% (95% CI: −4.16 to −1.81), reflecting a significant overall decline.

For NH Asian or Pacific Islander individuals, the AAMR decreased significantly from 2000 to 2011 (APC: −2.37%; 95% CI: −3.51 to −1.21), followed by a further significant decline from 2011 to 2016 (APC: −7.49%; 95% CI: −11.92 to −2.84), and then a significant increase from 2016 to 2020 (APC: 5.33%; 95% CI: 0.88 to 9.98). For the Asian subgroup reported separately from 2021 onward, the AAMR declined significantly from 10.20 in 2021 to 7.08 in 2024 (APC: −11.37%; 95% CI: −20.16 to −1.61). The overall AAPC for the Asian or Pacific Islander group (2000–2020) was −2.20% (95% CI: −3.64 to −0.74), a significant decline.

For Hispanic or Latino individuals, the AAMR declined significantly from 2000 to 2018 (APC: −2.47%; 95% CI: −2.89 to −2.05), then increased non‐significantly between 2018 and 2021 (APC: 10.41%; *p* = 0.059), followed by a significant decline through 2024 (APC: −12.38%; 95% CI: −16.72 to −7.82). The overall AAPC from 2000 to 2024 was −2.26% (95% CI: −3.59 to −0.92), a significant decline.

For NH American Indian or Alaska Native individuals, the AAMR showed a non‐significant increase from 2000 to 2002 (APC: 11.65%; *p* = 0.519), followed by a significant decline from 2002 to 2021 (APC: −1.35%; 95% CI: −2.16 to −0.53), and a non‐significant decline from 2021 to 2024 (APC: −8.74%; *p* = 0.115). The overall AAPC from 2000 to 2024 was −1.29% and was not statistically significant (95% CI: −4.32 to 1.82; *p* = 0.411), decreasing overall from 11.66 in 2000 to 8.05 in 2024.

**Figure 5:**
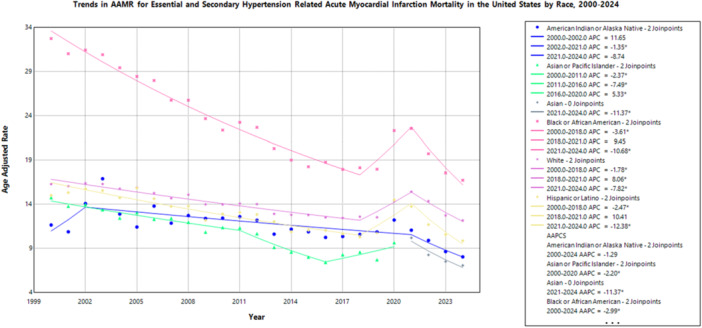
Trends in age‐adjusted mortality rates for essential and secondary hypertension‐related acute myocardial infarction in the United States by race, 2000–2024.

The AAMR for NH White individuals showed a significant overall decline from 2000 to 2024 (AAPC: −1.38%; 95% CI: −2.26 to −0.51), with a significant decline from 2000 to 2018 (APC: −1.78%; 95% CI: −2.00 to −1.55), a significant increase from 2018 to 2021 (APC: 8.06%; 95% CI: 0.96 to 15.65), and a significant decline from 2021 to 2024 (APC: −7.82%; 95% CI: −10.74 to −4.81), decreasing overall from 16.29 in 2000 to 12.17 in 2024 (Supporting Information S1: Table 6; Figure 5).

### 3.6 Crude Mortality Rates for Essential and Secondary Hypertension‐Related Myocardial Infarction Stratified by Age Group

When stratified by 10‐year age group, the CMR was highest among people aged 85+ years, followed by those aged 75–84 years, 65–74 years, 55–64 years, 45–54 years, 35–44 years and 25–34 years (overall CMR 85+ years: 161.9; 95% CI: 161.2–162.5; 75–84 years: 64.5; 95% CI: 64.3–64.8; 65–74 years: 30.1 (95% CI: 29.9–30.2), 55–64 years: 14.3 (95% CI: 14.2–14.4), 45–54 years: 4.9 (95% CI: 4.9–5.0), 35–44 years: 1.1 (95% CI: 1.1–1.1), and 25–34 years: 0.14 (95% CI: 0.13–0.14). In brief, the CMR for the 85+ year age group showed a significant overall decline from 198.1 to 122.8 over the study period (AAPC: −2.03%; 95% CI: −3.24 to −0.81). The CMR for the 75–84 years and the 65–74‐years age groups showed similar patterns with a significant decline initially (75–84 years: APC −2.74 from 2000 to 2018; 65–74 years: APC −2.47 from 2000 to 2013) followed by a significant increase between 2018 and 2021 (65–74 years: APC 7.15;) and a significant decrease afterward (65–74 years: APC −7.44 from 2021 to 2024).

The 55–64 age group showed more dynamic trends: a significant decline from 2000 to 2018 (APC: −0.59; 95% CI: −0.93 to −0.26), a borderline non‐significant increase from 2018 to 2021 (APC: 9.30; 95% CI: −0.21 to 19.72), followed by a significant decline again through 2024 (APC: −8.99; 95% CI: −13.08 to −4.71), whereas the 45–54 age group showed a modest but significant increase from 2000 to 2018 (APC: 0.39; 95% CI: 0.05–0.73), before following the same later pattern of a non‐significant rise to 2021 and significant decline to 2024.

**Figure 6:**
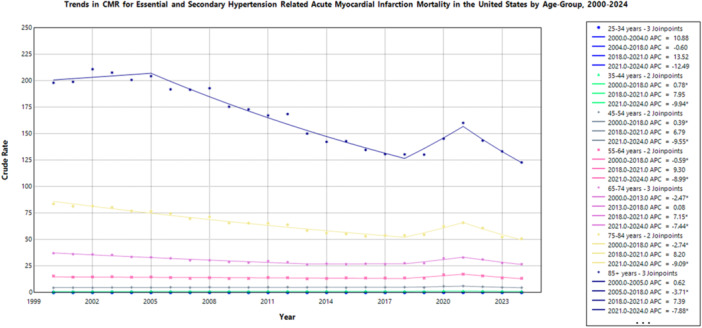
Trends in crude mortality rate for essential and secondary hypertension‐related acute myocardial infarction in the United States by age group, 2000–2024.

For the 35–44 years group, there was also a modest but significant increase from 2000 to 2018 (APC: 0.78; 95% CI: 0.23–1.34). The CMR for the 25–34 years age group showed no statistically significant trend at any point from 2000 to 2024 (overall AAPC: 1.29; 95% CI: 2.76–5.51), rising modestly from 0.09 to 0.13 per 100,000 (Supporting Information S1: Table 7; Figure 6).

### 3.7 Sub‐Group Analysis

**Figure 7:**
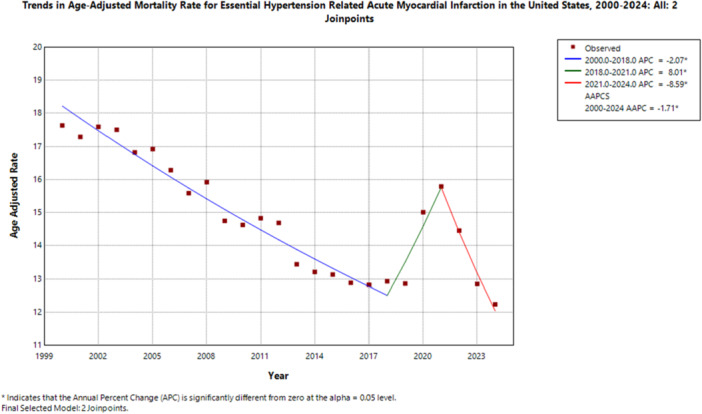
Overall age‐adjusted mortality rate for essential hypertension‐related acute myocardial infarction in the United States, 2000–2024.

For AMI deaths in which EH specifically was listed as a contributing cause, the AAMR was 17.63 in 2000 and declined significantly through 2018 (APC: −2.07%; 95% CI: −2.30 to −1.84; *p* < 0.000001). This was followed by a significant increase from 2018 to 2021, during which the AAMR rose to a peak of 15.79 (APC: 8.01%; 95% CI: 0.62 to 15.94; *p* = 0.035), before declining significantly again through 2024 to 12.24 (APC: −8.59%; 95% CI: −11.70 to −5.36; *p* < 0.001). The overall trend from 2000 to 2024 was a significant decline (AAPC: −1.71%; 95% CI: −2.62 to −0.79; *p* < 0.001) (Figure 7).

## 4 Discussion

A 25‐year mortality analysis using data from the CDC WONDER revealed significant trends in hypertension‐related AMI mortality. From 2000 to 2018, the AAMR declined steadily and was followed by a sharp increase from 2018 to 2021, before declining again from 2021 to 2024. Several clinical and systemic factors may help explain this variation. The decline in AMI mortality among hypertensive individuals across most of the study period indicates that improvements in primary prevention, emergency care, and continuing treatment have significantly improved the outlook for patients. Despite this, the AAMR rose sharply between 2018 and 2021, before declining again through 2024. Such variation may reflect stresses caused by the COVID‐19 pandemic and may highlight weaknesses in current healthcare systems [16, 17].

In line with previous literature, our findings confirm that males experience higher AAMRs than females. In 2021, AAMR for men was higher than that for females, and this difference remained evident across long‐term trends. Historical data on coronary artery disease show a similar pattern, suggesting that this disparity may stem from both biological sex differences and behavioral factors. Notably, women may present with atypical symptoms of MI, which can lead to delayed diagnosis and undertreatment, particularly among older women [18, 19]. Findings suggest that women who experience AMI are less likely to receive guideline‐recommended treatments, experience higher rates of post‐event heart failure, and are re‐hospitalized more frequently than men [20].

Inequalities in terms of ethnicity were identified; NH Black adults had the highest AAMR among all racial and ethnic groups examined. These trends align with previous studies, possibly because of differences in social determinants of health, access to and use of preventive care, financial status, and quality of healthcare treatment received, as well as chronic exposure to stressors that affect cardiovascular health [21, 22, 23].

State‐level disparities were evident, with the highest AAMR in the top 90th percentile states (Arkansas, Mississippi, and Tennessee). These states align with the historically defined “stroke belt” and “heart failure belt,” regions characterized by a combination of systemic factors—such as inadequate healthcare infrastructure, shortages of cardiologists, and entrenched lifestyle‐related risk factors [24].

South Dakota and Rhode Island, though outside this traditional belt, also ranked among the highest‐burden states. South Dakota's elevated AAMR likely reflects reduced healthcare access among its large rural and Native American population, along with rate volatility from its smaller size [25]. Rhode Island's burden appears driven more by hypertension control and poor medication adherence than regional access, both accelerating atherosclerosis and arrhythmic risk, with the burden compounded by co‐occurring diabetes and obesity [26].

Compared with metropolitan areas, non‐metropolitan areas presented higher mortality trends. These differences may be influenced by factors such as limited availability of high‐acuity cardiac care, longer travel distances to percutaneous coronary intervention (PCI) centers, and reduced access to early diagnostics and follow‐up care [27].

Among the different age groups, patients aged 85 years and older presented by far the highest CMR, consistent with the cumulative burden of cardiovascular risk factors with advancing age. Notably, however, the youngest age groups (25–34 and 35–44 years) did not show the same protective downward trend observed in older cohorts, which may reflect adults in these age groups beginning to accumulate cardiovascular risk factors earlier in life, mainly obesity, diabetes, and hypertension [28]. Such findings align with recent studies predicting a rising cardiovascular burden among younger populations [29].

Although hypertension is one of the most manageable cardiovascular risk factors, a significant percentage of individuals in the US still do not have it under adequate control. The temporary rise in mortality observed among people with hypertension during the 2018–2021 period highlights the challenges patients face in adhering to medication regimens, adjusting dosages when needed, and actively engaging in their treatment plans, particularly during periods of healthcare system strain [30]. In addition to clinical management, cardiovascular disease prevention must also address key lifestyle‐related risk factors, including poor diet, physical inactivity, and tobacco use—behaviors that are disproportionately prevalent among populations with lower socioeconomic status [31].

These mortality trends should be interpreted alongside national hypertension control trends. NHANES data indicate that the proportion of US adults with hypertension achieving blood pressure control rose substantially from 31.8% in 1999–2000% to 53.8% in 2013–2014, but this progress reversed in subsequent years, declining to approximately 48.2% by 2017–2020, with no further improvement through 2021–2023 [23, 32]. This decline in control was not uniform, disproportionately affecting older adults, women, and NH Black adults—groups that, notably, also showed elevated or persistent AMI mortality in the present analysis. The stagnation in hypertension control nationally may help explain why the protective downward trend in AMI mortality observed earlier in the study period failed to continue uniformly after 2018, particularly given that this plateau in blood pressure control coincided closely with the onset of the mortality increase identified in our Joinpoint analysis.

Finally, census region trends indicated that the South had the highest mortality, while the Northeast consistently had the lowest, throughout the study period. These differences likely reflect broader structural and socioeconomic inequities, including disparities in insurance coverage, healthcare access, and investment in preventive public health systems [33].

## 5 Study Limitations

Limitations of the study should be acknowledged. First, there is potential for data misrepresentation and omission because the study relies on ICD codes I10–I15 and I21 and death certificates. Second, the dataset lacked information on the health condition of the patients concerning their disease status. The dataset did not include the details of the clinical parameters or the treatment and management of the patients. Lastly, the dataset did not incorporate the socioeconomic status of the patients, which could affect their access to healthcare facilities.

## 6 Conclusion

To sum up, although the overall AAMR for AMI in hypertensive individuals in the US has declined over the study period, this decline has not been shared equally across population subgroups. Disparities by sex, race and ethnicity, geographic region, and urbanicity persisted throughout, and in some subgroups, mortality trends diverged further over time rather than converging. Addressing these disparities will require coordinated efforts among healthcare systems, policymakers, and public health stakeholders to target the upstream social and structural determinants of cardiovascular health. Continued surveillance using CDC WONDER and similar data platforms will be essential to monitoring progress and informing targeted interventions to reduce preventable AMI‐related mortality among individuals with hypertension.

## Author Contributions

**Javeria Akhter** and **Aasiya Shahbaz:** conceptualization, study design, data acquisition, data curation, formal statistical analysis, interpretation of results, drafting of the original manuscript, and critical revision of the manuscript for important intellectual content. **Waleed Ahmad** and **Aniqa Dejwani:** methodology development, interpretation of findings, clinical contextualization of results, and critical review and editing of the manuscript. **Nafila Zeeshan, Aasiya Shahbaz** and **Jamil Nasrallah:** literature review, validation of results, assistance in data interpretation, and manuscript review and editing. All authors reviewed and approved the final version of the manuscript and agree to be accountable for all aspects of the work.

## Funding

The authors have nothing to report.

## Ethics Statement

Due to the de‐identified nature, the need for Institutional Review Board approval is waived.

## Consent

Due to the de‐identified nature, the need for consent to participate is waived. The final draft of this work has been read and approved by all authors, who also consent to its publication.

## Conflicts of Interest

The authors declare no conflicts of interest.

## Supporting information


Supporting File

## Data Availability

All data utilized in this analysis are publicly available and have been sourced from publicly accessible CDC WONDER database.
