# Supplementary material for: Epidemiological Trends in Mortality From Acute Myocardial Infarction With Essential and Secondary Hypertension: CDC WONDER Database Analysis, 2000–2024
Source: Clin Cardiol. 2026 Jul 18;49(7):e70420. doi: 10.1002/clc.70420 (PMC13379940; doi:10.1002/clc.70420)
Supplement: Supplementary file 1 — Supporting File [file CLC-49-e70420-s001.docx]

**Supplementary Material**

**Supplementary Table 1.** Place of Death for Essential and Secondary Hypertension-Related mortality trends in Acute Myocardial Infarction in the United States, 2000-2024

| Place of Death | Deaths | % of Total Deaths |
| --- | --- | --- |
| Medical Facility - Inpatient | 215967 | 25.62% |
| Medical Facility - Outpatient or ER | 169369 | 20.09% |
| Medical Facility - Dead on Arrival | 12687 | 1.50% |
| Medical Facility - Status unknown | 399 | 0.047% |
| Decedent's home | 297972 | 35.36% |
| Hospice facility | 9624 | 1.14% |
| Nursing home/long term care | 104593 | 12.41% |
| Other | 30409 | 3.60% |
| Place of death unknown | 1635 | 0.19% |

**Supplementary Table 2.** Overall AAMR in Essential and Secondary Hypertension-Related mortality trends in Acute Myocardial Infarction in the United States, 2000-2024

| Year | Age Adjusted Mortality Rate | 95% Lower CI | 95% Upper CI |
| --- | --- | --- | --- |
| 2000 | 17.63 | 17.44 | 17.82 |
| 2001 | 17.29 | 17.1 | 17.48 |
| 2002 | 17.59 | 17.39 | 17.78 |
| 2003 | 17.5 | 17.31 | 17.69 |
| 2004 | 16.82 | 16.63 | 17 |
| 2005 | 16.92 | 16.74 | 17.11 |
| 2006 | 16.28 | 16.1 | 16.46 |
| 2007 | 15.6 | 15.43 | 15.77 |
| 2008 | 15.92 | 15.75 | 16.1 |
| 2009 | 14.75 | 14.59 | 14.91 |
| 2010 | 14.63 | 14.47 | 14.8 |
| 2011 | 14.83 | 14.67 | 15 |
| 2012 | 14.69 | 14.53 | 14.85 |
| 2013 | 13.44 | 13.29 | 13.59 |
| 2014 | 13.21 | 13.06 | 13.35 |
| 2015 | 13.13 | 12.99 | 13.28 |
| 2016 | 12.89 | 12.74 | 13.03 |
| 2017 | 12.83 | 12.69 | 12.97 |
| 2018 | 12.93 | 12.79 | 13.07 |
| 2019 | 12.86 | 12.73 | 13 |
| 2020 | 15.01 | 14.86 | 15.15 |
| 2021 | 15.79 | 15.64 | 15.95 |
| 2022 | 14.49 | 14.35 | 14.64 |
| 2023 | 12.85 | 12.71 | 12.98 |
| 2024 | 12.26 | 12.13 | 12.39 |

**Supplementary Table 3.** Overall Trend in Age-Adjusted Mortality Rates for Essential and Secondary Hypertension-Related mortality trends in Acute Myocardial Infarction by Sex in the United States, 2000-2024

| Year | Male | Female |
| --- | --- | --- |
| 2000 | 20.25 | 15.35 |
| 2001 | 20.05 | 14.9 |
| 2002 | 20.45 | 15.19 |
| 2003 | 20.57 | 14.86 |
| 2004 | 20.08 | 14.12 |
| 2005 | 19.9 | 14.33 |
| 2006 | 19.76 | 13.45 |
| 2007 | 18.66 | 12.94 |
| 2008 | 19.33 | 13.06 |
| 2009 | 18.09 | 11.99 |
| 2010 | 18.15 | 11.71 |
| 2011 | 18.49 | 11.78 |
| 2012 | 18.48 | 11.54 |
| 2013 | 16.93 | 10.49 |
| 2014 | 16.88 | 10.12 |
| 2015 | 16.67 | 10.09 |
| 2016 | 16.52 | 9.79 |
| 2017 | 16.59 | 9.6 |
| 2018 | 16.9 | 9.55 |
| 2019 | 16.95 | 9.42 |
| 2020 | 19.76 | 10.96 |
| 2021 | 20.73 | 11.58 |
| 2022 | 19.2 | 10.47 |
| 2023 | 16.89 | 9.44 |
| 2024 | 16.22 | 8.86 |

**Supplementary Table 4.** Overall Trend in Age-Adjusted Mortality Rates for Essential and Secondary Hypertension-Related mortality trends in Acute Myocardial Infarction by States in the United States, 2000-2024

| State | Age-Adjusted Mortality Rate | 95% Lower Confidence Interval | 95% Upper Confidence Interval |
| --- | --- | --- | --- |
| Alabama | 11.57 | 11.28 | 11.85 |
| Alaska | 6.82 | 6.03 | 7.65 |
| Arizona | 10.25 | 10.00 | 10.51 |
| Arkansas | 36.82 | 36.12 | 37.53 |
| California | 15.76 | 15.63 | 15.88 |
| Colorado | 9.05 | 8.77 | 9.33 |
| Connecticut | 7.60 | 7.36 | 7.84 |
| Delaware | 14.23 | 13.49 | 15.00 |
| District of Columbia | 14.71 | 13.71 | 15.75 |
| Florida | 10.16 | 10.03 | 10.29 |
| Georgia | 12.64 | 12.39 | 12.89 |
| Hawaii | 9.48 | 9.03 | 9.93 |
| Idaho | 18.48 | 17.77 | 19.20 |
| Illinois | 14.20 | 14.01 | 14.39 |
| Indiana | 15.36 | 15.06 | 15.65 |
| Iowa | 13.66 | 13.30 | 14.03 |
| Kansas | 9.02 | 8.67 | 9.37 |
| Kentucky | 23.20 | 22.72 | 23.70 |
| Louisiana | 15.51 | 15.13 | 15.90 |
| Maine | 12.43 | 11.87 | 13.00 |
| Maryland | 16.90 | 16.58 | 17.22 |
| Massachusetts | 8.96 | 8.76 | 9.16 |
| Michigan | 12.28 | 12.08 | 12.47 |
| Minnesota | 8.46 | 8.22 | 8.70 |
| Mississippi | 37.58 | 36.81 | 38.37 |
| Missouri | 15.30 | 15.02 | 15.58 |
| Montana | 7.73 | 7.18 | 8.30 |
| Nebraska | 9.11 | 8.68 | 9.55 |
| Nevada | 7.32 | 6.95 | 7.70 |
| New Hampshire | 9.71 | 9.23 | 10.20 |
| New Jersey | 13.38 | 13.16 | 13.59 |
| New Mexico | 10.05 | 9.62 | 10.47 |
| New York | 11.97 | 11.82 | 12.11 |
| North Carolina | 15.26 | 15.01 | 15.51 |
| North Dakota | 15.33 | 14.54 | 16.15 |
| Ohio | 19.06 | 18.83 | 19.28 |
| Oklahoma | 14.40 | 14.05 | 14.76 |
| Oregon | 11.71 | 11.39 | 12.05 |
| Pennsylvania | 14.21 | 14.03 | 14.40 |
| Rhode Island | 21.56 | 20.81 | 22.34 |
| South Carolina | 20.38 | 19.96 | 20.80 |
| South Dakota | 24.68 | 23.59 | 25.81 |
| Tennessee | 21.33 | 20.97 | 21.70 |
| Texas | 17.11 | 16.95 | 17.29 |
| Utah | 7.69 | 7.30 | 8.08 |
| Vermont | 14.13 | 13.30 | 14.99 |
| Virginia | 11.40 | 11.16 | 11.64 |
| Washington | 12.93 | 12.67 | 13.20 |
| West Virginia | 18.76 | 18.20 | 19.32 |
| Wisconsin | 16.89 | 16.56 | 17.21 |
| Wyoming | 15.19 | 14.17 | 16.26 |

**Supplementary Table 5.** Age-Adjusted Mortality Rates for Essential and Secondary Hypertension-Related mortality trends in Acute Myocardial Infarction by Census Region in the United States, 2000-2024

| **Year** | **Northeast** | | **Midwest** | | **South** | | **West** | |
| --- | --- | --- | --- | --- | --- | --- | --- | --- |
|  | **AAMR** | **95% CI** | **AAMR** | **95% CI** | **AAMR** | **95% CI** | **AAMR** | **95% CI** |
| **2000** | 16.53 | 16.12–16.94 | 19.03 | 18.62–19.45 | 18.22 | 17.89–18.56 | 16.15 | 15.73–16.57 |
| **2001** | 16.60 | 16.19–17.00 | 18.22 | 17.81–18.62 | 17.79 | 17.46–18.11 | 16.09 | 15.68–16.50 |
| **2002** | 16.65 | 16.24–17.06 | 18.27 | 17.87–18.67 | 18.20 | 17.88–18.53 | 16.82 | 16.41–17.24 |
| **2003** | 15.85 | 15.46–16.25 | 17.96 | 17.57–18.36 | 18.66 | 18.33–18.99 | 16.68 | 16.26–17.09 |
| **2004** | 15.54 | 15.15–15.93 | 17.02 | 16.64–17.40 | 17.78 | 17.46–18.09 | 16.30 | 15.90–16.70 |
| **2005** | 15.05 | 14.67–15.43 | 17.38 | 17.00–17.76 | 18.26 | 17.94–18.58 | 15.93 | 15.54–16.32 |
| **2006** | 14.32 | 13.95–14.69 | 17.00 | 16.62–17.37 | 17.23 | 16.92–17.53 | 15.91 | 15.52–16.29 |
| **2007** | 13.67 | 13.31–14.03 | 16.15 | 15.78–16.51 | 16.64 | 16.34–16.94 | 14.99 | 14.62–15.36 |
| **2008** | 13.91 | 13.56–14.27 | 16.37 | 16.00–16.73 | 16.97 | 16.67–17.27 | 15.61 | 15.24–15.99 |
| **2009** | 12.56 | 12.22–12.89 | 15.27 | 14.92–15.62 | 16.15 | 15.86–16.44 | 13.96 | 13.62–14.31 |
| **2010** | 12.49 | 12.15–12.82 | 14.88 | 14.53–15.22 | 15.99 | 15.71–16.27 | 14.11 | 13.76–14.46 |
| **2011** | 12.94 | 12.60–13.28 | 15.48 | 15.14–15.83 | 15.85 | 15.57–16.13 | 14.16 | 13.82–14.50 |
| **2012** | 13.02 | 12.68–13.36 | 15.09 | 14.75–15.43 | 15.84 | 15.56–16.11 | 13.86 | 13.52–14.19 |
| **2013** | 11.64 | 11.32–11.96 | 13.84 | 13.52–14.17 | 14.73 | 14.47–14.99 | 12.49 | 12.18–12.80 |
| **2014** | 11.64 | 11.32–11.96 | 13.83 | 13.51–14.15 | 14.50 | 14.24–14.75 | 11.85 | 11.55–12.14 |
| **2015** | 11.29 | 10.98–11.60 | 13.64 | 13.32–13.95 | 14.43 | 14.18–14.68 | 11.92 | 11.63–12.22 |
| **2016** | 10.84 | 10.54–11.15 | 13.03 | 12.72–13.34 | 14.29 | 14.04–14.53 | 12.15 | 11.85–12.44 |
| **2017** | 10.55 | 10.25–10.84 | 13.25 | 12.94–13.56 | 14.30 | 14.05–14.54 | 11.83 | 11.54–12.11 |
| **2018** | 10.42 | 10.13–10.71 | 13.23 | 12.92–13.53 | 14.72 | 14.47–14.96 | 11.73 | 11.45–12.01 |
| **2019** | 10.36 | 10.07–10.65 | 12.72 | 12.43–13.02 | 14.92 | 14.67–15.16 | 11.60 | 11.32–11.88 |
| **2020** | 12.23 | 11.91–12.54 | 14.26 | 13.95–14.57 | 17.85 | 17.58–18.11 | 13.20 | 12.91–13.49 |
| **2021** | 11.61 | 11.30–11.92 | 14.89 | 14.57–15.22 | 19.44 | 19.16–19.72 | 13.94 | 13.64–14.25 |
| **2022** | 10.92 | 10.63–11.22 | 13.72 | 13.42–14.03 | 17.51 | 17.25–17.77 | 13.01 | 12.73–13.30 |
| **2023** | 9.63 | 9.36–9.90 | 12.00 | 11.72–12.29 | 15.86 | 15.62–16.10 | 11.30 | 11.03–11.57 |
| **2024** | 9.22 | 8.96–9.48 | 11.33 | 11.06–11.61 | 15.28 | 15.04–15.51 | 10.51 | 10.26–10.76 |

**Supplementary Table 6.** Overall Trend in Age-adjusted Mortality Rates for Essential and Secondary Hypertension-Related mortality trends in Acute Myocardial Infarction by Urbanization and Race in the United States, 2000-2024

| Year | Metropolitan | Non-metropolitan | American Indian or Alaska Native | Asian or Pacific Islander | Black or African-American | White | Hispanic or Latino |
| --- | --- | --- | --- | --- | --- | --- | --- |
| 2000 | 17.30 | 19.52 | 11.66 | 14.75 | 32.74 | 16.29 | 15.01 |
| 2001 | 16.94 | 19.24 | 10.89 | 13.78 | 31.02 | 16.06 | 15.33 |
| 2002 | 17.10 | 20.42 | 14.09 | 13.98 | 31.43 | 16.39 | 15.75 |
| 2003 | 16.86 | 20.96 | 16.9 | 13.39 | 30.91 | 16.3 | 15.56 |
| 2004 | 16.18 | 20.37 | 12.9 | 12.44 | 29.43 | 15.76 | 14.74 |
| 2005 | 16.15 | 21.06 | 11.43 | 13.07 | 28.45 | 15.91 | 15.86 |
| 2006 | 15.57 | 20.24 | 13.8 | 12.3 | 27.99 | 15.27 | 14.64 |
| 2007 | 14.82 | 19.70 | 11.87 | 12.42 | 25.75 | 14.69 | 13.76 |
| 2008 | 15.12 | 20.35 | 12.73 | 11.97 | 25.75 | 15.1 | 13.8 |
| 2009 | 13.80 | 19.79 | 12.4 | 10.85 | 23.67 | 14 | 12.19 |
| 2010 | 13.74 | 19.43 | 12.44 | 11.37 | 22.37 | 13.98 | 12.91 |
| 2011 | 13.95 | 20.02 | 12.59 | 11.33 | 23.25 | 14.09 | 13.08 |
| 2012 | 13.83 | 20.03 | 12.2 | 10.68 | 22.68 | 14.02 | 12.85 |
| 2013 | 12.60 | 18.50 | 10.62 | 9.13 | 20.31 | 12.92 | 12.05 |
| 2014 | 12.31 | 19.00 | 11.18 | 8.59 | 19.01 | 12.82 | 10.89 |
| 2015 | 12.07 | 19.51 | 10.89 | 8.02 | 18.26 | 12.79 | 11.07 |
| 2016 | 11.90 | 19.22 | 10.25 | 7.43 | 18.77 | 12.52 | 11.05 |
| 2017 | 11.76 | 19.53 | 10.36 | 8.3 | 17.96 | 12.47 | 10.66 |
| 2018 | 11.83 | 20.02 | 10.59 | 8.59 | 18.14 | 12.6 | 10.28 |
| 2019 | 11.77 | 20.21 | 10.87 | 7.72 | 17.98 | 12.53 | 10.78 |
| 2020 | 13.54 | 24.55 | 12.22 | 9.65 | 22.32 | 14.43 | 14.5 |
| 2021 |  |  | 11.07 | 10.2 | 22.55 | 15.43 | 13.75 |
| 2022 |  |  | 9.91 | 8.27 | 19.73 | 14.36 | 11.7 |
| 2023 |  |  | 8.65 | 7.51 | 17.56 | 12.75 | 10.57 |
| 2024 |  |  | 8.05 | 7.08 | 16.73 | 12.17 | 9.87 |

**Supplementary Table 7.** Crude Mortality Rates for Essential and Secondary Hypertension-Related mortality trends in Acute Myocardial Infarction by Age-group in the United States, 2000-2024

| Ten-Year Age Groups | Crude Death Rate | 95% Confidence Interval |
| --- | --- | --- |
| Young population | | |
| 25-34 years | 0.14 | 0.10 – 0.17 |
| 35-44 years | 1.08 | 0.98 – 1.17 |
| 45-54 years | 4.94 | 4.73 – 5.15 |
| 55-64 years | 14.30 | 13.93 – 14.71 |
| Older population | | |
| 65-74 years | 30.06 | 29.93 – 30.20 |
| 75-84 years | 64.54 | 64.27 – 64.80 |
| 85+ years | 161.86 | 161.19 – 162.52 |
